# Lineages of *Streptococcus equi* ssp. *equi* in the Irish equine industry

**DOI:** 10.1186/2046-0481-66-10

**Published:** 2013-06-04

**Authors:** Emma Moloney, Kerrie S Kavanagh, Tom C Buckley, Jakki C Cooney

**Affiliations:** 1 Department of Life Sciences, University of Limerick, Limerick, Ireland; 2 Irish Equine Centre, Johnstown, Naas, Co. Kildare, Ireland; 3 Materials and Surface Sciences Institute, University of Limerick, Limerick, Ireland

**Keywords:** *Streptococcus equi* ssp. *equi*, Strangles, *seM*

## Abstract

**Background:**

*Streptococcus equi* ssp. *equi* is the causative agent of ‘Strangles’ in horses. This is a debilitating condition leading to economic loss, yard closures and cancellation of equestrian events. There are multiple genotypes of *S. equi* ssp. *equi* which can cause disease, but to date there has been no systematic study of strains which are prevalent in Ireland. This study identified and classified *Streptococcus equi* ssp. *equi* strains isolated from within the Irish equine industry.

**Results:**

Two hundred veterinary isolates were subjected to SLST (single locus sequence typing) based on an internal sequence from the *seM* gene of *Streptococcus equi* ssp *equi*. Of the 171 samples which successfully gave an amplicon, 162 samples (137 Irish and 24 UK strains) gave robust DNA sequence information. Analysis of the sequences allowed division of the isolates into 19 groups, 13 of which contain at least 2 isolates and 6 groups containing single isolates. There were 19 positions where a DNA SNP (single nucleotide polymorphism) occurs, and one 3 bp insertion. All groups had multiple (2–8) SNPs. Of the SNPs 17 would result in an amino acid change in the encoded protein. Interestingly, the single isolate EI8, which has 6 SNPs, has the three base pair insertion which is not seen in any other isolate, this would result in the insertion of an Ile residue at position 62 in that protein sequence. Comparison of the relevant region in the determined sequences with the UK *Streptococcus equi seM* MLST database showed that Group B (15 isolates) and Group I (2 isolates), as well as the individual isolates EI3 and EI8, are unique to Ireland, and some groups are most likely of UK origin (Groups F and M), but many more probably passed back and forth between the two countries.

**Conclusions:**

The strains occurring in Ireland are not clonal and there is a considerable degree of sequence variation seen in the *seM* gene. There are two major clades causing infection in Ireland and these strains are also common in the UK.

## Background

*Streptococcus equi* ssp. *equi*, which is a biovar of *S. zooepidemicus,* is the causative agent of ‘Strangles’ in horses. This devastating condition is highly contagious, has high morbidity, and can lead to stable closures. Outbreaks result in significant disruption of equestrian events and economic loss. Although the complete mechanism for pathogenesis is not understood, the role of the M protein (SeM) has been proposed in virulence (see review [1]). SeM is a surface bound protein which diminishes phagocytosis of the bacterial cell by inhibiting deposition of C3b on the cell surface [2]. The protein has also been shown to bind fibrinogen, and IgG [3].

The *seM* gene is present in the majority of strains but, analogous to the *emm* genes of *S. pyogenes*, is highly divergent. This property has allowed use of the *seM* sequence as a means for discriminating strains. Multi-locus typing of *S. equi* ssp. *equi* does not produce a significant advantage over single-locus typing based on *seM*, and studies in the UK have shown that based on the *seM* gene sequence analysis outbreak specific strains can be identified [4-6].

‘Strangles’ is a significant problem in the Irish equine sector. Data obtained from the Animal Health Trust, UK, suggest that annually there are dozens of outbreaks reported annually in Ireland (data obtained from the International Collating Centre, based at the Animal Health Trust, UK). However, there is a paucity of information about the diversity of Irish strains. This is the first thorough investigation of the strains causing ‘Strangles’ outbreaks in Ireland. The results show that there are a diverse range of strains in the Irish equine population, some of which appear to be unique to Ireland. However, several frequently isolated Irish strains were related to strains isolated from large outbreaks in the UK.

## Results

### Amplification and sequence determination

An amplicon of 661 bp (Additional file 1: Figure S1 lane 1) was obtained for the *seM* locus from 171 strains, indicting the presence of the *seM* gene in these isolates. Amplicons were not obtained from all test strains, indicting the *seM* gene was either absent or had significant sequence divergence in the priming regions for these strains. These isolates were excluded from further analysis. An amplicon of 694 bp Additional 1: Figure S1. lane 2 was obtained for the *sclC* locus from a limited set of 47 strains.

### Grouping of Irish strains

Initial analysis of the *seM* amplicon sequences identified 20 variations of the sequence compared to the Se4047 type strain sequence. Many Irish isolates had more than one mutation when compared to the type strain. Table 1 shows the location and type of the particular mutations for representative isolates and the number of strains harbouring that particular combination of mutations. Additional file 2: Table S1 provides information on the strain composition of the groups as well as the year of isolation and the location in Ireland where the strain was isolated. The most populated four groups contain 79% of all isolates analysed, and are therefore the most likely strains to have caused outbreaks during the sampling time. Interestingly, there were also 4 Irish groups which contained only a single isolate, which would suggest that these are possible sources of new outbreak strains.

**Table 1:** Single nucleotide polymorphisms (SNPs) associated with individual groups in this study

**Base number**		**32**	**37**	**123**	**169**	**174**	**179**		**184**	**185**	**187**	**191**	**193**	**268**	**274**	**312**	**319**	**323**	**350**	**379**	**391**
Se4047		G	A	T	A	A	C		A	G	A	A	G	C	T	T	G	A	C	G	G
**Group**	**Total isolates (Irish, UK)**																				
A	11 (8, 3)	*	*	*	*	*	*		*	*	*	*	*	T	*	*	A	*	*	*	*
B	15 (15, 0)	*	*	*	*	*	*		*	*	G	*	*	T	*	*	A	G	*	*	*
C	41 (34, 7)	*	*	*	*	*	*		*	*	*	*	*	T	*	*	A	G	*	*	*
D	4 (4, 0)	*	*	*	*	T	*		G	A	*	*	*	*	*	C	A	G	*	A	*
E	6 (6, 0)	*	*	*	*	*	*		*	*	*	*	A	T	*	*	A	G	*	*	*
F	2 (0, 2)	*	*	*	*	G	G		*	*	*	*	*	*	*	*	A	*	*	*	*
G	57 (52, 5)	*	*	*	*	T	*		G	A	*	*	*	*	*	C	A	*	*	A	*
H	7 (6, 1)	*	*	*	*	G	*		G	*	*	G	*	*	*	C	A	*	*	A	*
I	2 (2, 0)	*	*	*	G	*	*		*	*	*	*	*	T	*	*	A	G	*	*	*
J	2 (1, 1)	*	*	*	*	*	*		G	*	*	*	*	*	*	C	A	*	*	A	*
K	4 (4, 0)	T	*	*	*	T	*		G	A	*	*	*	*	*	C	A	*	*	A	*
L	3 (3, 0)	*	G	*	*	T	*		G	A	*	*	*	*	*	C	A	*	*	A	*
M	2 (0, 2)	*	*	*	*	G	G		*	*	*	*	*	*	*	*	A	*	*	*	A
**Single isolates**																					
EI3	1 (1, 0)	*	*	*	*	T	*		G	A	*	*	*	*	*	C	A	*	T	A	*
EI4	1 (1, 0)	*	*	*	*	*	*		*	A	G	*	*	T	*	*	A	G	T	A	*
EI5	1 (1, 0)	T	*	*	*	*	*		*	*	*	*	*	T	*	*	A	G	*	*	*
EI8	1 (1, 0)	*	*	*	*	T	*	ATA^a^	G	A	*	*	*	*	*	C	A	*	*	A	*
UK11	1 (0 , 1)	*	*	*	*	T	*		*	*	*	*	*	*	C	*	A	*	*	*	*
UK21	1 (0, 1)	*	*	A	*	T	*		G	A	*	*	*	*	*	C	A	G	*	A	*
Se4047 residue		Ser	Arg	Ser	Ser	Glu	Ala		Ser		Arg	Asp	Ala	Leu	Ser	Asn	Val	His	Pro	Ala	Val
SNP generated residue		Ile	Gly	Arg	Gly	Asp	Gly	Ile	Asn, Asp, Gly^b^		Gly	Gly	Thr	Phe	Pro	n/c^c^	Met	Arg	Leu	Thr	Ile

*Base the same as the type strain sequence. ^a^ Insertion occurs between base 182 and 183 in the type strain sequence. ^b^ SNPs at 184 and 185 affect the same codon. ^c^ No change in sequence.

Analysis of DNA sequences of an amplified region of the *sclC* was initially included in this study in an attempt to broaden the analysis scheme. However, sequence analysis of the 47 *sclC* amplicons showed no heterogeneity in sequence. This strongly suggests that this gene is not a good target for discriminating between Irish strains, and the study was limited to 50 isolates. It should be noted that these 50 isolates had a diverse range of *seM* sequences, indicating the strains were not identical.

### Comparison to the UK PubMLST database of *seM* types

The sequences obtained in this study were compared to sequences in the online resource the PubMLST *Streptococcus equi seM* database (http://pubmlst.org/perl/mlstdbnet/agdbnet.pl?file=sz_seM.xml). It should be noted that the amplicons in this study are not identical to those used in the PubMLST *seM* database. This study is based on sequence from base 15–557 of the *seM* gene. The *seM* database uses bases 112–438 of *seM*, which is entirely contained within the region sequenced in this study (bases 15–557). As a result, some of the unique groups identified in this study belong to the same *seM* grouping according to the UK database. There are novel SNPs found in Groups K, and L, and isolate EI5 at positions 32 and 37, which encode amino acids within the signal sequence of SeM and are outside the SeM database target sequence (Table 1). This will result in mutations Ser11Ile and Arg13Gly, respectively, in the protein sequence when compared to the Se4047 SeM sequence. Sequence corresponding to region 112–438 of *seM* from all groups or individual strains in this study were submitted to the PubMLST *seM* database and the allele identified as the nearest hit is tabulated in Table 2. With some exceptions (outlined below) each study group *seM* had identity over the common sequence with strains associated with outbreaks in the UK, and Sweden. One group (Group B) had identity with an *seM* allele (allele 17) which is a strain that was not associated with an outbreak. Group I, and isolates EI4 and EI8 did not have any match to sequences in the UK database.

**Table 2:** **Identification of nearest nucleotide allele match in *seM *database for strains of *Streptococcus equi *used in this project**

**Group**	**Nucleotide allele match in *seM *MLST database**	**Number of recorded UK isolates**	**Outbreak years**
A	7	33	2004-2010
B	17	0	
C	6	103	2003-2010
D	48	4	2006-2010
E	59	8	2007-2010
F	16	26	2006-2008
G	9	161	2003-2010
H	46	13	2006-2010
I	Nearest allele 6 - Identity 99.69% Differences: 1: nt 58: A → G		
J	51	8	2007-2010
K	9	161	2003-2010
L	9	161	2003-2010
M	44	46	2006-2007
**Single isolates**			
EI3	52	3	2008-2010
EI4	Nearest allele 17 - Identity 99.08% Differences: 3: nt 74: G → A, nt 239: C → T, nt 268: G→ A		
EI5	6	103	2003-2010
EI8	Partial match: allele 69 – Identity 99.09% Gaps: 1, three base insertion		
UK11	8	3	1999-2003
UK21	68	1	2003
Se4047	3	4	1990-2008

### The Irish strains belong to two major clades

**Figure 1:**
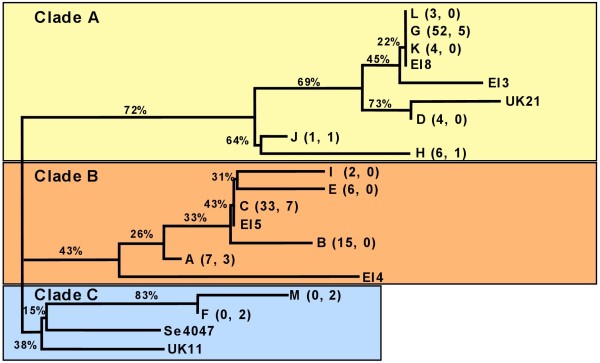
**Cladogram of *S. equi *ssp. *equi seM *sequences using a Neighbour joining method, with 1000 bootstraps.**The diagram indicates the division of the strains into three major clades based on alignments of the sequence of *seM* amplicon. The names of the groups are the same as those given in Table 1. For multi-strain groups the number of strains present in each group is given in parenthesis (Irish strains, UK strains). Irish and UK strains that are not identical to any other isolate are indicated with EI and UK respectively followed by a strain number. The percentages given on the horizontal lines are the bootstrap values for the adjacent branch point on the right.

Phylogenetic analyses of the sequences were performed. The first analysis was based on nucleotides 112–438 of *seM*. The phylogenetic tree is presented in Figure 1. A similar analysis with the extended 15–557 sequences was also performed (data not presented). Both analyses resulted in the strains grouping in to three clades (indicated as Clades A, B and C in Figure 1). The majority of the sequences were contained in two major clades (A and B). These two clades contain all the Irish sequences as well as UK strains, while Clade C is comprised of groups F and M, strain UK11 and the reference strain Se4047 which are exclusively strains of UK origin. The group composition of the clades did not vary between the two analyses, however the extended sequence did allow finer discrimination between groups L, G, K and strain EI8.

The two major clades (A and B) are roughly associated with the two major groups, Group G (Clade A) and Group C (Clade B). The definitive difference between these two branches is the presence of a G at position 184 in all Clade A isolates and a T at position 268 in all Clade B isolates. These mutations are unique to these clades. Clade C is characterized by having fewer mutations than the other clades and having a unique G residue at position 179, however strain UK11 does not have this mutation.

In addition, all sequences generated in this study were compared in a phylogenetic analysis with all 128 *seM* allelic variants available at the PubMLST database. This data is presented as Additional file 3: Figure S2. The branches containing sequences from this study are highlighted by red annotation. The Irish strains are represented in two major divisions in the cladogram, marked Regions 1 and 2. Although this study has limitations due to restricted sample size, it is of note that Irish strains are not dispersed throughout the entire tree, but are distinctly clustered.

## Discussion

Prior to this study there was very limited information available on the diversity of Irish *S. equi* ssp. *equi* strains, and how these strains are related to strains from the UK. This study establishes that, despite conservation seen in the *sclC* gene, Irish strains are not clonal and that the *seM* gene was shown to be divergent in sequence from the type strain (Se4047) at nineteen locations, with an additional 3 bp sequence insertion present in one isolate. A given isolate can have more than one of these changes, and there were nineteen distinct combinations of SNP found. The strains were categorized into unique groups based on a distinct pattern of SNPs. The majority of these groups contained multiple isolates.

A Neighbour-joining cladogram constructed with the extended and MLST based *seM* sequences showed that the Irish strains could be divided into two major clades (Clades A and B) Figure 1. These clades contained both Irish and UK derived sequences. The third clade (Clade C) contained six sequences, all of UK origin. With one exception (Group J), all Clade A strains have six or more SNPs, while with one exception (isolate EI4) all Clade B and Clade C strains have 4 or less SNPs. This finding suggests that the Clade A represents a distinct genetic/clonal population with a higher innate recombination frequency. Studies have established that the 5’ region of the *emm* gene in *Streptococcus pyogenes* is more susceptible to evolutionary pressure [7]. However, because the branch pathway to the main node of Clade A is longer than that of other clades, it may also represent an older clade in which sequence substitutions have accumulated.

There is a predominant cluster of mutations between residues 169 and 193 accounting for nine of the twenty sequences differences identified in this study. This cluster comprises eight SNPs, and in one case, strain E8, a codon is inserted between residue 182 and 183. At the protein level these changes will affect the N-terminal region of the mature protein. In fact, all but one of the mutations (T312C) will result in a change of protein sequence. This is consistent with the findings of Ijaz and colleagues, who demonstrated that there is increased antigenic drift in the SeM protein when compared to other *S. equi* surface proteins [8]. The binding of Fibrinogen (Fg) by SeM has been mapped to a broad region of the amino acid sequence (residues 37 to 352, corresponding to bases 111 and 1056), while amino acid residues 71 to 421 (bases 213 to 1263) are associated with Ig binding [3]. Interestingly, this cluster of mutations (between bases 169 and 193) is in a region of the protein uniquely associated with the Fg binding region. Variation in the N-terminal sequence of M proteins is well described and has been suggested to be part of a mechanism for antigenic variation [7]. The variation in M protein sequence does not automatically result in a loss of biological function [9-11]. However, detailed biochemical studies would be required to determine if the sequence changes seen in this study influence the level or specificity of Fg binding.

The *seM* DNA sequences were examined for relatedness to *seM* from known isolates. The sequence analysed in this study is longer than that employed in the UK PubMLST *Streptococcus equi seM* database, 543 bases compared with 327 bases. However, the 327 base sequence is completely contained within the target sequence in this study. There is additional flanking sequence information provided in this study, which has resulted in a higher level of discrimination between closely related strains. Some of the Irish and UK strains (*e.g.* Group G, K and L strains) are therefore identical to the same allele in the *seM* MLST database but are clearly distinguishable in this analysis. The overall outcome of this analysis is that the majority of Irish strains from this study have a counterpart in the UK, and these strains have been involved in significant outbreaks across Ireland and the UK. In the case of the smaller isolate groups (*e.g.* Groups D, E and L) containing only Irish strains, there are recorded outbreaks of strains with identical *seM* alleles in the UK. The lack of UK isolates in these groups in this study could be as a result of the small sample size of UK strains used herein. However, strains causing major outbreaks in Ireland (Groups C and G) appear to be responsible for major outbreaks in the UK (allele 6 and allele 9 respectively, causing 103 and 161 outbreaks respectively in the UK). Interestingly, there is one significant group (Group B), comprising 15 strains all of Irish origin, which has no counterpart in the UK database. These isolates have been identified since 2006, and 13 of the 15 isolates in this study come from 2008 and 2009. In addition, Group I and isolates EI4 and EI8 have unique sequences not identified in the UK database. These strains may represent Irish derived strains. The Clade C isolates are all UK derived strains and are associated with significant outbreaks (Group F, 26 recorded outbreaks, and Group M 46 recorded outbreaks). However, the possibility that they occur in Ireland can not be precluded by this study. This clade contains a unique SNP (C179G) which does not occur in the Irish strains. This could be useful in tracking new outbreaks in Ireland and their origins.

For the most part, this study shows there is considerable overlap between Irish and UK *S. equi* ssp. *equi* outbreak strains. The strains causing major outbreaks in Ireland appear to be those responsible for major outbreaks in the UK. Given the nature of the exchange between Ireland and the UK this is not surprising. Monitoring dissemination of country specific strains will provide useful information on the movement of animals, the ability to trace outbreaks and their origins, and ultimately may aid improvement of quarantine procedures.

## Conclusions

The strains occurring in Ireland are not clonal and there is a considerable degree of sequence variation seen in the *seM* gene. The study has shown that the majority of isolates belong to two major clades and these clades contain both Irish and UK strains. It is therefore likely that there is considerable exchange of strains between Ireland and the UK. However, there is also evidence to suggest that location specific isolates occur and more extensive monitoring of these types of strains will provide information on the movement of strains between the two islands.

## Methods

### Bacterial strains and culture

Samples for bacterial culture were plated onto Columbia Blood agar, Columbia Blood agar with Streptococcus supplement (Oxoid), and Wilkins Chalgren agar. The plates were incubated aerobically and anaerobically at 37°C. After incubation, the plates were examined for *S. equi* ssp. *equi* colonies, sub-cultured for purity and incubated for a further 24 hours. API Strep strips (BioMerieux) and carbohydrate fermentation tests were carried out to confirm the identity of the isolates. Strains were preserved by freezing at −80°C in 30% glycerol.

A total of two hundred isolates were obtained from the Irish Equine Centre strains collection and 30 isolates obtained from The Animal Health Trust in Newmarket, Suffolk. All Irish isolates were from veterinary sources from ‘Strangles’ outbreaks in Ireland collected between 2006 and 2009 by the Irish Equine Centre.

Isolate *Se*4047 from Hampshire, New Forest in England, isolated in 1990 by Animal Health Trust was used as the reference strain and used for comparison purposes for the *seM* and *sclC* genes in this project. This reference strain was the focus of the *S. equi* ssp. *equi* genome-sequencing project at the Sanger Institute [12].

### Preparation of template DNA for PCR analysis

Bacteria were revived from storage and subcultured on 5% (v/v) defibrinated sheep blood (Charles River Laboratories) incorporated into Todd Hewitt (TH) agar (Sigma-Aldrich, Ireland). The plates were incubated overnight at 37°C in a static air incubator.

A single colony of *S. equi* ssp. *equi* from a freshly cultured plate was suspended in 10 mL TH broth and incubated for 16 hr at 37°C. Cells from 1.5 mL were harvested by centrifugation 2 min at 12,000 x g. The culture medium was removed and the pellet was resuspended in 200 μL of Gram-positive lysis solution (GenElute kit, Sigma-Aldrich, Ireland) containing 250 units/mL mutanolysin and 2 x 10^6^ units/mL lysozyme. The mixture was incubated at 37°C for 30 min. Further purification involved the GenElute Bacterial Genome kit, which was used as per manufacturer’s instructions. Purified DNA was stored at −20°C until required.

### PCR amplification of *seM* and *sclC* gene fragments for sequence analysis

Specific regions of the *seM* and *sclC* genes were amplified from genomic DNA of *S. equi* ssp. *equi* using primers seM_F and seM_R for the *seM* amplicon, and sclC_F and sclC_R for the *sclC* amplicon [13]. Sequences of the primers are presented in Table 3. Thirty-five cycles of amplification were performed used *Taq* polymerase (Invitrogen, UK) and an annealing temperature of 55°C. PCR products were analysed by agarose gel electrophoresis. Multiple reactions were performed for each sample yielding approximately 2 μg of DNA.

**Table 3:** **Oligonucleotide Primers used in the PCR study of *Streptococcus equi *ssp. *equi***

**Primer**	**Sequence**	**Location**	**Product Size (bp)**
*seM_*F	GTACTGCATAAAGAAGTTCCTGTC	83 bp 5’ to *seM*	661
*seM_*R	GATTCGGTAAGAGCTTGACGCTCATC	nt 578	
*sclC_* F	ACCAGCCAGCAGCACTAAAATAT	nt 113	694
*sclC*_R	GGCTGCTTTTTGACCTGTTGGT	nt 807	

For each *S. equi* ssp. *equi* isolate, peak PCR fractions were pooled and purified using QIAquick spin columns (Qiagen, UK) according to manufacturer’s instructions. Samples and primers for sequencing were sent to Eurofins, MWG Operon (Ebersberg, Germany). Samples for sequencing contained 70 μL at 20 ng DNA/μL.

### DNA sequence analysis

The quality of the DNA sequences was assessed by manual inspection of the sequence chromatogram. Sequences not providing a good quality of reaction over the entire length of the sequence were withheld from further analysis. Sequences were aligned against the known *seM* sequence of the reference strain *S. equi* ssp. *equi Se*4047, using T-Coffee (T-Coffee Multiple Sequence Alignment Tool [14]). Sequence differences were collated in a database, highlighting SNPs and Indels (insertions and deletions), and the residue position with respect to the reference strain.

The corresponding amino acid sequences, determined from representative unique DNA sequences, were aligned with the protein sequence of SeM from strain *Se*4047 using T-Coffee.

### Phylogenetic analysis

Multiple nucleotide and protein sequences were aligned using Clustal-W and T-Coffee [14,15]. These programs calculate the best match and show the differences and similarities between the selected sequences entered.

Phylogenetic and molecular evolutionary analyses were conducted using the phylip output from T-Coffee and genetic distance based neighbour-joining algorithms [16] using the Kimura 2 parameters at the TRex online server. Bootstrap analysis was performed to estimate the confidence of the tree topology [17].

## Competing interests

The authors declare that they have no competing interests.

## Authors’ contributions

EM carried out the molecular genetic studies, participated in the sequence alignment and helped to draft the manuscript. KS carried out strain databanking and initial isolate typing. TB conceived of the study, and participated in its design and helped to draft the manuscript. JC conceived of the study, and participated in the design and analysis and drafted the manuscript. All authors read and approved the final manuscript.

## Supplementary Material

Additional file 1: Figure S1 Analysis of PCR amplicons for *seM* and *sclC* from *S. equi* ssp. *equi***.** Lane 1 contains the *seM* amplicon, lane 2 contains the *sclC* amplicon. The lane marked M contains the size markers (1 kb plus, Invitrogen UK). Click here for file

Additional file 2: Table S1 *Streptococcus equi* ssp. *equi* Irish strains used in this study. Click here for file

Additional file 3: Figure S2 Integrated phylogentic analysis of study strains with the *Streptococcus equi seM* MLST database strains.The alignment for construction of the tree was performed using T-Coffee and the Phylip output used to construct the tree using neighbour joining methods. Branches containing strains from this study are indicated by annotating text in red. For clarity, the image should be viewed at 200% magnification or printed on A3 paper. Click here for file
