# Supplementary material for: Lineages of Streptococcus equi ssp. equi in the Irish equine industry
Source: Ir Vet J. 2013 Jun 4;66(1):10. doi: 10.1186/2046-0481-66-10 (PMC3679875; doi:10.1186/2046-0481-66-10)
Supplement: Additional file 1: Figure S1 — Analysis of PCR amplicons for seM and sclC from S. equi ssp. equi. Lane 1 contains the seM amplicon, lane 2 contains the sclC amplicon. The lane marked M contains the size markers (1 kb plus, Invitrogen UK). [file 2046-0481-66-10-S1.doc]

**Figure S1**

**
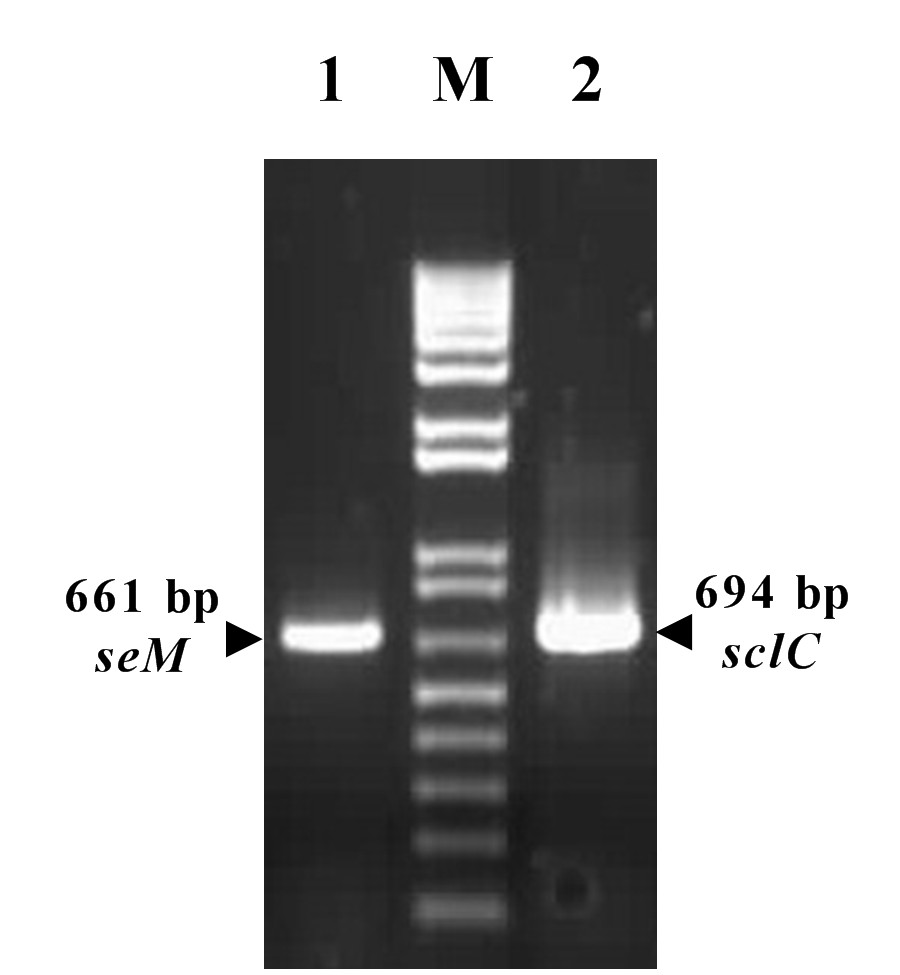
**

**Figure S1. Analysis of PCR amplicons for *seM* and *sclC*  from *S. equi* ssp. *equi*.**

Lane 1 contains the *seM*  amplicon, lane 2 contains the *sclC* amplicon. The lane marked M contains the size markers (1 kb plus, Invitrogen UK).
