# Supplementary material for: Lineages of Streptococcus equi ssp. equi in the Irish equine industry
Source: Ir Vet J. 2013 Jun 4;66(1):10. doi: 10.1186/2046-0481-66-10 (PMC3679875; doi:10.1186/2046-0481-66-10)
Supplement: Additional file 2: Table S1 — Streptococcus equi ssp. equi Irish strains used in this study. [file 2046-0481-66-10-S2.doc]

**Table S1. *Streptococcus equi* ssp. *equi* Irish strains used in this study.**

| **Strain** | **Year** | **Location** | **Group** |
| --- | --- | --- | --- |
| 08M13228 | 2008 | Carlow | C |
| 08M5102 | 2008 | Carlow | D |
| 06M10322 | 2006 | Kildare | G |
| 06M10701 | 2006 | Kildare | C |
| 06M10827 | 2006 | Kildare | C |
| 06M12095 | 2006 | Limerick | I |
| 06M12276 | 2006 | Kildare | C |
| 06M13164 | 2006 | Kildare | C |
| 06M13244 | 2006 | Wicklow | G |
| 06M13245 | 2006 | Cork | C |
| 06M13425 | 2006 | Cork | C |
| 06M13437 | 2006 | Cork | C |
| 06M13973 | 2006 | Kildare | G |
| 06M18837 | 2006 | Galway | G |
| 06M18974 | 2006 | Meath | G |
| 06M19433 | 2006 | Tipperary | G |
| 06M19712 | 2006 | Cork | C |
| 06M19882 | 2006 | Kildare | B |
| 06M20419 | 2006 | Kildare | J |
| 06M20565 | 2006 | Kildare | C |
| 06M20891 | 2006 | Meath | C |
| 06M7126 | 2006 | Kildare | C |
| 06M7920 | 2006 | Kildare | C |
| 06M8449 | 2006 | Meath | G |
| 07L798 | 2007 | n/a^*^ | L |
| 07M10149 | 2007 | NI^**^ | H |
| 07M10255 | 2007 | NI^**^ | H |
| 07M15311 | 2007 | NI^**^ | EI5 |
| 07M15395 | 2007 | Wicklow | G |
| 07M15429 | 2007 | Tipperary | K |
| 07M16120 | 2007 | NI^**^ | K |
| 07M16226 | 2007 | Kildare | K |
| 07M16227 | 2007 | n/a | G |
| 07M16228 | 2007 | Kildare | G |
| 07M16626 | 2007 | Limerick | G |
| 07M17854 | 2007 | Cork | C |
| 07M18857 | 2007 | Donegal | G |
| 07M19313 | 2007 | Wexford | C |
| 07M19901 | 2007 | Monaghan | G |
| 07M20582 | 2007 | Tipperary | C |
| 07M20621 | 2007 | Kildare | C |
| 07M20645 | 2007 | Kildare | L |
| 07M21680 | 2007 | Clare | G |
| 07M21833 | 2007 | Cork | I |
| 07M23797 | 2007 | Meath | G |
| 07M4206 | 2007 | Wexford | G |
| 07M8238 | 2007 | Kildare | C |
| 07M8495 | 2007 | Laois | B |
| 07M8496 | 2007 | Kerry | D |
| 07M8635 | 2007 | Laois | G |
| 08M10521 | 2008 | Kildare | C |
| 08M10661 | 2008 | Meath | E |
| 08M110 | 2008 | Meath | G |
| 08M1117 | 2008 | Kildare | G |
| 08M12496 | 2008 | Cavan | E |
| 08M12821 | 2008 | Cork | G |
| 08M12912 | 2008 | Meath | G |
| 08M1295 | 2008 | Cork | H |
| 08M13340 | 2008 | Meath | A |
| 08M13343 | 2008 | Meath | A |
| 08M13536 | 2008 | Offaly | G |
| 08M13537 | 2008 | Galway | G |
| 08M13590 | 2008 | Kildare | C |
| 08M13653 | 2008 | Kildare | G |
| 08M13705 | 2008 | Meath | C |
| 08M14186 | 2008 | Kildare | C |
| 08M14741 | 2008 | Kildare | C |
| 08M15075 | 2008 | Wicklow | G |
| 08M15076 | 2008 | Tipperary | G |
| 08M15085 | 2008 | Limerick | H |
| 08M15174 | 2008 | Meath | C |
| 08M15467 | 2008 | Cork | G |
| 08M15946 | 2008 | Limerick | G |
| 08M16031 | 2008 | Meath | B |
| 08M16032 | 2008 | Meath | G |
| 08M16033 | 2008 | Meath | L |
| 08M16069 | 2008 | Longford | K |
| 08M17226 | 2008 | Dublin | G |
| 08M18 | 2008 | Dublin | A |
| 08M18121 | 2008 | Clare | D |
| 08M18164 | 2008 | Tipperary | C |
| 08M18847 | 2008 | Tipperary | G |
| 08M18860 | 2008 | Wexford | B |
| 08M1945 | 2008 | Mayo | G |
| 08M1961 | 2008 | Wexford | G |
| 08M20000 | 2008 | Kildare | G |
| 08M20203 | 2008 | Wexford | B |
| 08M20204 | 2008 | Wexford | EI4 |
| 08M20205 | 2008 | Wexford | B |
| 08M20331 | 2008 | Kildare | A |
| 08M20953 | 2008 | Kildare | B |
| 08M21576 | 2008 | Tipperary | B |
| 08M21760 | 2008 | Kildare | G |
| 08M22545 | 2008 | Clare | G |
| 08M3538 | 2008 | Clare | G |
| 08M3539 | 2008 | Clare | G |
| 08M362 | 2008 | Kerry | G |
| 08M3690 | 2008 | Meath | B |
| 08M5210 | 2008 | Meath | EI3 |
| 08M5445 | 2008 | Kildare | C |
| 08M5768 | 2008 | Wexford | A |
| 08M5978 | 2008 | Tipperary | C |
| 08M6975 | 2008 | Meath | D |
| 08M741 | 2008 | Galway | C |
| 08M79 | 2008 | Wexford | EI8 |
| 08M868 | 2008 | Cork | C |
| 08M9809 | 2008 | Kildare | C |
| 09M06113 | 2009 | Limerick | B |
| 09M10134 | 2009 | Kildare | B |
| 09M10134 | 2009 | Kildare | G |
| 09M11053 | 2009 | Kildare | G |
| 09M11218 | 2009 | Meath | G |
| 09M11387 | 2009 | Wexford | G |
| 09M12848 | 2009 | Laois | G |
| 09M12988 | 2009 | Kildare | G |
| 09M141 | 2009 | Kildare | A |
| 09M14416 | 2009 | Laois | H |
| 09M14435 | 2009 | Kildare | G |
| 09M14482 | 2009 | Tipperary | C |
| 09M14580 | 2009 | Meath | G |
| 09M14625 | 2009 | n/a^*^ | G |
| 09M14694 | 2009 | Limerick | E |
| 09M15320 | 2009 | Kildare | E |
| 09M15584 | 2009 | Tipperary | G |
| 09M16202 | 2009 | Kildare | G |
| 09M1994 | 2009 | Kildare | A |
| 09M4768 | 2009 | Limerick | B |
| 09M4841 | 2009 | Cork | H |
| 09M6870 | 2009 | Kilkenny | G |
| 09M7213 | 2009 | Meath | G |
| 09M7254 | 2009 | Limerick | B |
| 09M7865 | 2009 | Cork | E |
| 09M817 | 2009 | Laois | E |
| 09M8477 | 2009 | Meath | C |
| 09M8478 | 2009 | Meath | B |
| 09P139 | 2009 | n/a^*^ | B |
| m13519 | n/a^*^ | Tipperary | C |

^*^ Year or source location of isolate not available

^**^ Isolate from Northern Ireland
