# Supplementary material for: Lineages of Streptococcus equi ssp. equi in the Irish equine industry
Source: Ir Vet J. 2013 Jun 4;66(1):10. doi: 10.1186/2046-0481-66-10 (PMC3679875; doi:10.1186/2046-0481-66-10)
Supplement: Additional file 3: Figure S2 — Integrated phylogentic analysis of study strains with the Streptococcus equi seM MLST database strains.The alignment for construction of the tree was performed using T-Coffee and the Phylip output used to construct the tree using neighbour joining methods. Branches containing strains from this study are indicated by annotating text in red. For clarity, the image should be viewed at 200% magnification or printed on A3 paper. [file 2046-0481-66-10-S3.doc]

**Figure S2**

**
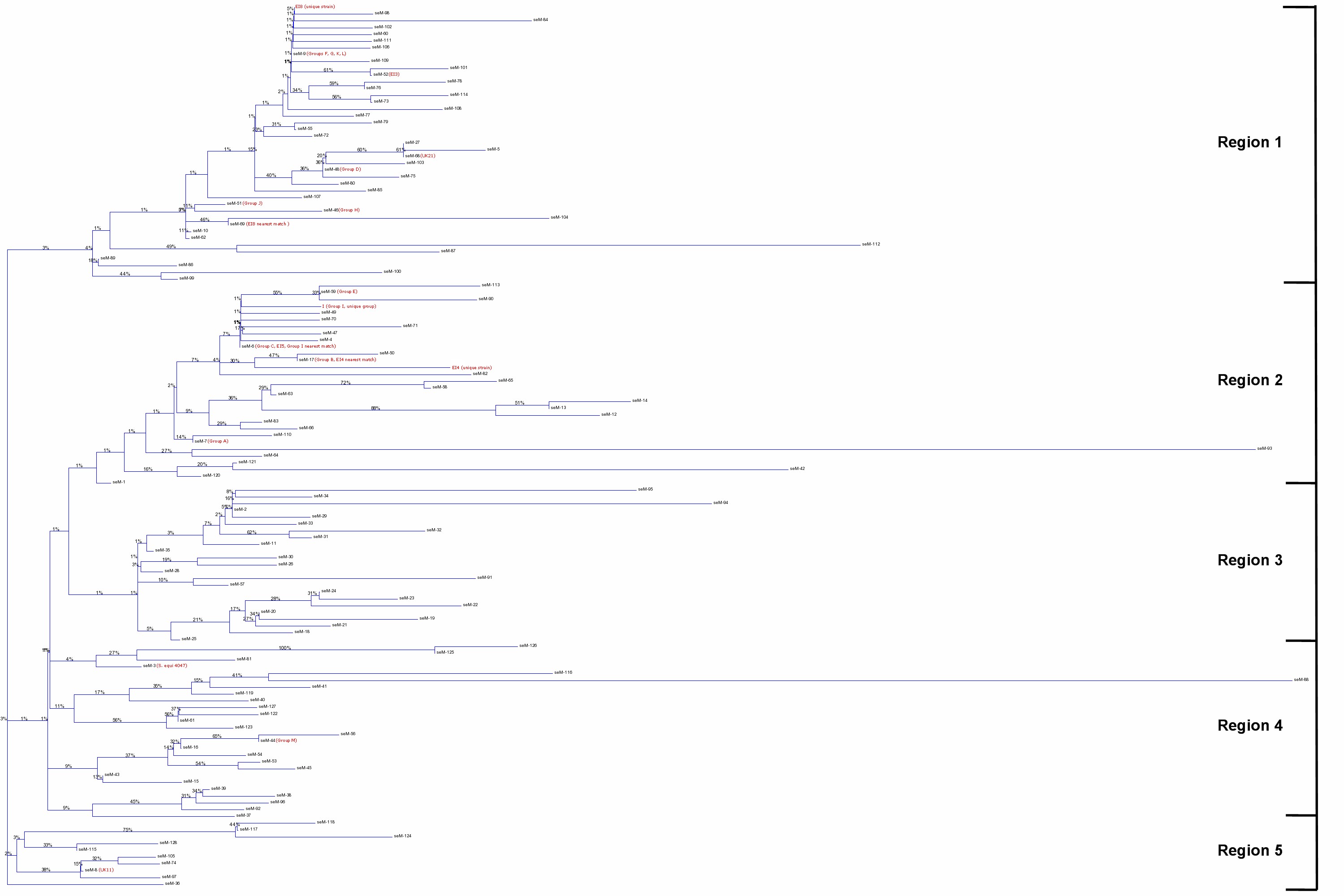
**

**Figure S2. Integrated phylogentic analysis of study strains with the *Streptococcus equi seM* MLST database strains.**

The alignment for construction of the tree was performed using T-Coffee and the Phylip output used to construct the tree using neighbour joining methods.

Branches containing strains from this study are indicated by annotating text in red. For clarity, the image should be viewed at 200% magnification or printed on A3 paper.
